# Towards Personalized Medicine Based on Platelet Function Testing for Stent Thrombosis Patients

**DOI:** 10.1155/2012/617098

**Published:** 2012-12-25

**Authors:** Thea Cornelia Godschalk, Christian Marcus Hackeng, Jurriën Maria ten Berg

**Affiliations:** St. Antonius Center for Platelet Function Research, St. Antonius Hospital, Koekoekslaan 1, 3435 CM Nieuwegein, The Netherlands

## Abstract

Stent thrombosis (ST) is a severe and feared complication of coronary stenting. Patients who have suffered from ST are usually treated according to the “one-size-fits-all” dosing regimen of aspirin and clopidogrel. Many ST patients show high on-treatment platelet reactivity (HPR) despite this antiplatelet therapy (APT). It has been shown that HPR is a risk factor for major adverse cardiac events. Therefore, ST patients with HPR are at a high risk for recurrent atherothrombotic events. New insights into the variable response to clopidogrel and the advent of stronger P2Y12 inhibitors prasugrel and ticagrelor have changed the attention from a fixed APT treatment strategy towards “personalized APT strategies.” Strategies can be based on platelet function testing, which gives insight into the overall response of a patient to APT. At our outpatient ST clinic, we practice personalized APT based on platelet function testing to guide the cardiologist to a presumed optimal antiplatelet treatment of ST patients. Beside results of platelet function testing, comedication, clinical characteristics, and genetics have to be considered to decide on personalized APT. Ongoing studies have yet to reveal the optimal personalized APT strategy for cardiologists to prevent their patients from atherothrombotic and bleeding events.

## 1. Introduction

Dual antiplatelet therapy (DAPT) with aspirin and a P2Y12 inhibitor (clopidogrel, prasugrel, or ticagrelor) is the standard treatment to prevent atherothrombotic events in patients undergoing percutaneous coronary intervention (PCI) with stent implantation. Despite optimal therapy, 1–4% of these patients develop coronary stent thrombosis (ST) [1–7]. ST is a feared complication as it results in myocardial infarction in up to 80% and mortality in up to 40% of the cases. It also has a high recurrence rate (5–36%) [1, 3–6, 8, 9].

The pathophysiology and underlying mechanisms of ST are multifactorial. One of the most important risk factors for ST is the cessation of clopidogrel within the first six months after stent implantation [2, 5, 10–12], partly caused by noncompliance of the patient to DAPT. Other risk factors can be divided into clinical (e.g., diabetes mellitus, younger age), procedural (e.g., bifurcation stenting, undersizing of placed stent), angiographic (e.g., multivessel disease), and genetic factors (CYP2C19*2/*3) [2, 3, 5, 13–20]. Recently, paraoxonase-1 was identified as an important enzyme for the bioactivation of clopidogrel. Individuals homozygous for the loss-of-function allele of paraoxonase-1 (PON1 192QQ) had lower plasma levels of clopidogrel and were at higher risk for stent thrombosis [21]. However, this strong association could not be confirmed by other studies [22].

Recent studies have shown that a lower degree of platelet inhibition despite treatment with aspirin and clopidogrel, referred to as high on-treatment platelet reactivity (HPR), is also a risk factor for the occurrence of ST [23–33]. HPR in spite of clopidogrel use is shown in up to 30% of the western population and attributed to different clinical, pharmacological, and genetic factors [7, 34–38]. HPR is related to atherothrombotic events [7, 36, 39–46], and therefore monitoring of HPR has gained extensive attention [47]. 

Patients who have suffered from ST and are at high risk of atherothrombotic events including recurrent ST are usually treated with a fixed dose of aspirin and clopidogrel according the “one-size-fits-all” approach. With the release of the new P2Y12 inhibitors prasugrel and ticagrelor, more alternatives became available to combat HPR for clopidogrel and to tailor antiplatelet therapy (APT) strategies in patients with HPR. Platelet function tests aim to have the ability to differentiate between patients with HPR and no HPR and can, therefore, serve as a base to tailor APT [7, 30, 48]. Since many ST patients suffer from HPR, this group of patients might especially benefit from personalized medicine instead of the commonly used “one-size-fits-all” dosing regimen of DAPT in preventing the atherothrombotic events.

Hence, the objective of this paper is to discuss the importance and practice of personalized APT based on platelet function testing for stent thrombosis patients and to introduce our approach to counter HPR in ST patients at our outpatient stent thrombosis clinic.

## 2. Definitions of Stent Thrombosis

The presentation of ST differs in the level of documentation and timing and is categorized by these features by the Academic Research Consortium (ARC; Table 1). ST is called “definite” when confirmed with angiography or pathology, “probable” in the case of unexplained death within 30 days after PCI, and “possible” in the case of any unexplained death after 30 days following PCI. The timing of ST can be classified as acute (≤24 hours), subacute (>24 hours to ≤30 days), late (>30 days to ≤1 year), and very late (>1 year) ST [49]. The former two are often referred as early onset ST and the latter two as late onset ST. 

## 3. Platelet Function Tests

Multiple platelet function tests are available to measure the inhibitory effects of aspirin and P2Y12 inhibitors on platelet function and to correlate the extent of residual platelet reactivity to the occurrence of atherothrombotic and bleeding events. Cut-off values are often based on the receiver-operator characteristic (ROC) curve analysis, dividing patients in two groups: patients with a normal on-treatment platelet reactivity (NPR) and patients with HPR. The main characteristics in which platelet function tests differ are the pathways of thrombus formation that are measured, whether plasma or whole blood is used and whether the test is easy to use or labor intensive.

### 3.1. Light Transmittance Aggregometry

The classical platelet function test, often referred to as the “golden standard,” is light transmittance aggregometry (LTA). This test is based on the optical detection of aggregation in platelet-rich plasma. In two separate centrifuge steps platelet-rich plasma (PRP) and platelet-poor plasma (PPP) are obtained from citrated whole blood. A light beam is passed through the samples, whereby the amount of light transmitted through PPP is defined as 100% and through PRP as 0% aggregation. Several agonists such as arachidonic acid (AA) and adenosine diphosphate (ADP) are used to TRIGGER platelet aggregation in the samples. The formation of platelet aggregates will change the optical density in the samples which is a measure for the percentage of aggregation. Multiple studies have demonstrated that HPR measured with AA- and ADP-induced LTA correlates to adverse outcomes [7, 28, 36, 40, 50]. However, LTA needs intensive labor, is hardly standardized between laboratories, and is difficult to reproduce. Therefore, point-of-care platelet function tests are developed to overcome these difficulties. 

### 3.2. VerifyNow

The VerifyNow System (Accumetrics, San Diego, CA, USA) is a citrated whole blood assay designed to measure the agonist-induced platelet aggregation by turbidimetric based optical detection. It is a cartridge-based method to determine the response to aspirin and P2Y12 inhibitors. Platelets are activated by the presence of agonists and bind to fibrinogen-coated beads, after which the agglutinates fall out of the solution. The difference in optical density is measured. For the aspirin assay, the agonist AA is used and the result is expressed as aspirin reaction units (ARUs). The P2Y12 assay consists of two chambers with agonists which are used to determine the response to P2Y12 inhibitors. In the first chamber, the agonist ADP/antagonist prostaglandin E1 (PGE1) are used, and in the second chamber, the agonist TRAP is used to approximate a baseline for the maximal off-drug platelet reactivity. Results are reported as P2Y12 reaction units (PRU), a BASE value, and a percentage of inhibition calculated from the BASE and PRU. The VerifyNow assay is a typical point-of-care test as it is easy to use and results are quickly available and reproducible. Results of the assays correlate very well with LTA [51].

### 3.3. Vasodilator-Stimulated Phosphoprotein

The vasodilator-stimulated phosphoprotein (VASP) is an intraplatelet actin regulatory protein. Activation of the P2Y12 receptor by ADP causes dephosphorylation of VASP and activation of the glycoprotein IIb/IIIa receptor on the surface of the platelet, the main receptor for platelet adhesion and aggregation [52]. Conversely, inhibition of the P2Y12 receptor induces phosphorylation of VASP (VASP-P). VASP-P state reflects the extent of P2Y12 inhibition.

Flow cytometric analysis of the VASP-P state is performed using a standardized diagnostic assay kit (PLT VASP/P2Y12-assay from BioCytex, Marseille, France) [53]. Citrated blood is incubated with PGE1 or PGE1+ADP and fixed with paraformaldehyde after which the platelets are permeabilized and immunolabeled using a CD61 phycoerythrin-labeled platelet-specific antibody and a FITC-labeled VASP-P-specific mouse monoclonal antibody or a negative isotopic control antibody. Platelets are identified by its forward and side scatter distribution using the flow cytometer. A platelet reactivity index (PRI) is calculated from the corrected mean fluorescence intensity (MFIc): PRI (%) = [(MFIc PGE1 − MFIc (PGE1+ADP))/MFIc PGE1] × 100. A high PRI represents poor platelet inhibition by the P2Y12 inhibitor. The PLT VASP/P2Y12 assay has demonstrated good correlation with LTA [54].

### 3.4. Platelet Function Analyzer

The Platelet Function Analyzer (PFA-100, Dade Behring, Germany) measures platelet function in citrated whole blood under high shear conditions of 4000 to 5000 sec^−1^ [55]. The time needed to form a platelet plug occluding the aperture cut into a membrane coated with agonists is determined and reported as closure time (CT) in seconds. The results are inversely related to platelet reactivity. If no significant platelet plug can be formed, the result is reported as >300 seconds. The membrane coated with collagen/epinephrine (Col/EPI) is sensitive to aspirin and the membrane coated with collagen/ADP (Col/ADP) is not sufficiently sensitive to P2Y12 inhibitors. For the Col/ADP cartridge, no correlation could be found between HPR and the outcome [7]. The test is easy to learn and semiautomatic. 

### 3.5. Multiplate

The Multiplate Analyzer (Dynabyte, Germany) is based on a hirudin anticoagulated whole blood multiple electrode aggregometry (MEA) and detects the increase in the electrical impedance resulting from the adhesion and aggregation of platelets on two independent sensor units in the test cuvette [56]. This increase in impedance is transformed to and reported as arbitrary aggregation units (AU) that are plotted against time (AU*min). Different agonists are available to monitor the effects of APT. The ASPI test is sensitive for aspirin and the ADP test is sensitive for P2Y12 inhibitors. The test needs only small amounts of blood, although some pipetting is required.

### 3.6. Thromboelastography

The thromboelastograph (TEG) is a function test that provides a global overview of hemostatic function, allowing insights into the interactions between the platelet and coagulation phase during blood clot formation as well as fibrinolysis properties. Citrated whole blood is placed into a cylindrical cup that oscillates back and forward. A stationary pin is immersed into the blood. The torque of the rotating cup is transmitted to the pin only when a blood clot linked the cup and pin together. The formation of the clot, the clot strength, and the clot lysis are measured over time and different parameters can be calculated. For example, the r-time represents the time elapsed from the start of the test to initial fibrin formation (velocity of thrombin generation) and the maximum amplitude (MA) represents the ultimate strength of the clot. MA and r-time seem to be predictive for adverse events [28]. 

## 4. High On-Treatment Platelet Reactivity and Stent Thrombosis

Multiple studies have shown an association between HPR and major adverse cardiac events (MACEs), including ST [7, 25, 36, 39–44]. Prospective studies have demonstrated an association of HPR with early onset ST [23, 25–27, 29, 33]. Geisler and coworkers [27] included 1,019 patients in a consecutive cohort study. Residual platelet aggregation was measured at least six hours after loading a dose of 600 mg clopidogrel and a followup for definite, probable, and possible ST was collected until three months after PCI. Residual platelet aggregation was an independent predictor of ST after three months. However, subgroup analysis showed only an association with the early onset ST (HR 1.05, *P* < 0.01), but not the late onset ST group. In a consecutive cohort by Sibbing and coworkers [33], response to clopidogrel for 1,608 patients who underwent PCI was determined by MEA. The upper quintile was defined as low response to clopidogrel. This low-responder group showed an association with the occurrence of ST until six months after stent implantation. Nevertheless, the majority of ST occurred within the first 30 days after PCI. Another study by Buonamici and coworkers [25], based on a sample of 804 patients who underwent PCI with drug eluting stent (DES) implantation, showed that nonresponders to clopidogrel, determined with LTA, had had an almost 4-fold increased risk to develop definite or probable ST until six months after DES implantation compared to clopidogrel responders. No comparison was made between early and late onset ST. Lev and co-workers [29] showed that patients with subacute ST had a similar platelet reactivity profile as patients presenting with STEMI but without ST. They questioned whether residual platelet reactivity despite DAPT really was a risk factor for subacute ST or was just associated with the acute setting of myocardial infarction. However, retrospective studies, measuring residual platelet reactivity in a stable phase more than 30 days after ST, also showed that HPR was associated with early onset ST [24, 30, 31, 57].

Beside HPR for clopidogrel, also dual HPR for clopidogrel and aspirin is associated with mainly early onset ST [26, 30, 58]. The largest retrospective study so far from Bouman and coworkers [24] included 84 patients with a history of ST and 74 control patients who had PCI with stent implantation but did not develop ST. Blood was drawn for platelet function testing before and after loading of both 600 mg clopidogrel and 100 mg aspirin. A heightened on-clopidogrel platelet reactivity was seen in the ST group compared to controls (42% versus 19%), and in the early onset ST group, HPR for clopidogrel was even seen in almost two-third of the patients. High on-aspirin platelet reactivity was seen in both the early and late onset ST groups compared to controls. HPR for both aspirin and clopidogrel was also more seen in both ST groups compared to controls.

In conclusion, these studies have shown that HPR is clearly associated with the occurrence of ST, however, also have shown that a large proportion of patients who have experienced ST have HPR. 

## 5. Personalized Medicine

The “one-size-fits-all” approach originated from key trials that have demonstrated that the combination of aspirin and clopidogrel significantly reduced MACE compared to aspirin therapy alone [59–63]. This approach is questioned more and more due to evidence of interindividual responses to clopidogrel, evidence that HPR is associated with MACE and with the recent advent of the more potent platelet inhibitors prasugrel and ticagrelor. Therefore, personalized APT strategies have become of particular interest.

With the introduction of the new antiplatelet agents prasugrel and ticagrelor, cardiologists do have more possibilities to tailor APT for their patients. Prasugrel and ticagrelor are both stronger P2Y12 inhibitors than clopidogrel and lower platelet reactivity more effectively [64, 65]. Both antiplatelet agents showed a significant reduction in ischemic events, but also showed increased bleeding events compared to clopidogrel [66, 67]. As a result, patients that respond well to clopidogrel will be on increased risk for bleeding complication when switched to prasugrel or ticagrelor. Therefore, efforts should be made to distinguish clopidogrel-treated patients that respond well to clopidogrel from patients that show HPR and will have a need for stronger P2Y12 inhibitors. 

Until today, no well-studied and accepted strategy is available for personalized antiplatelet treatment. The first study that reported on personalized APT based on platelet function testing was performed by Bonello and coworkers [44]. One hundred sixty-two patients with silent ischemia, stable angina, or non-ST elevated myocardial infarction (non-STEMI) were included. HPR was determined by VASP and patients were randomized to the control group that underwent PCI directly or to the VASP-guided group that received up to three additional loading doses of 600 mg clopidogrel until a PRI < 50% was established (which could not be achieved in 14% of the patients) before PCI. At one-month followup, MACE was significantly lower in the VASP-guided group than the control group, and no significant difference was seen in major and minor bleedings. However, definitive conclusions could not be drawn from this study due to the relatively small sample size and low event rate. The GRAVITAS study (gauging responsiveness with a VerifyNow assay-impact on thrombosis and safety) studied whether clinical outcome after stenting was improved after change of APT based on platelet function testing for HPR on clopidogrel [68]. Patients with HPR after receiving DES were randomized to a total first-day loading dose of 600 mg and a high dose of 150 mg clopidogrel per day or to no additional loading dose and the standard dose of 75 mg clopidogrel per day. Patients receiving 150 mg clopidogrel did not benefit from this strategy regarding the occurrence of cardiovascular death, MI, or ST. Possible reasons for these results were the limited effect in overcoming HPR by the double dose of clopidogrel and a low event rate [69]. In the TRIGGER-PCI study (testing platelet reactivity in patients undergoing elective stent placement on clopidogrel to guide alternative therapy with prasugrel), patients were randomized to 75 mg clopidogrel daily or prasugrel 10 mg daily when they exhibited HPR [70]. Prasugrel showed to effectively overcome HPR; however, due to the choice of a very low-risk group of patients with stable CAD with a very low event rate, the clinical benefit of personalized APT based on platelet function testing was not demonstrated in this study, which was prematurely halted. In accordance with the TRIGGER-PCI, other studies showed that HPR could be defeated with a change to another P2Y12 inhibitor in an all-comers population [71, 72]. Sambu and coworkers [73] demonstrated the feasibility to overcome HPR with personalized APT, especially in ST patients. Thirty-nine patients with ST were analyzed for HPR with a modified TEG and APT was adapted until NPR was shown. No recurrent ST was seen in this group; however, this study was not designed and powered to conclude on improvement of followup. The above mentioned studies could not demonstrate a convincing improvement in clinical outcome after APT adjustment in patients with HPR.

In conclusion, the personalized medicine based on platelet function testing does not seem to improve clinical outcome in low-risk groups. However, it can be hypothesized that patients at high risk for atherothrombotic events are more likely to benefit from tailored-based antiplatelet treatment strategies [49, 70]. Therefore, to practice personalized APT, especially for ST patients, we opened an outpatient stent thrombosis clinic.

## 6. Practice: Outpatient Stent Thrombosis Clinic

The objective of our outpatient stent thrombosis clinic (ST clinic) is to decrease atherothrombotic events by diminishing HPR but without exposing ST patients to increased bleeding risk. Therefore, HPR is determined on individual basis, after which patients who exhibit HPR are switched to an alternative APT regimen.

Patients with a history of ST are invited to our ST clinic, 30–60 days after the acute phase of ST. Blood samples are drawn for platelet function testing. Multiple platelet function tests are performed including LTA using the agonists AA and ADP, VerifyNow P2Y12 and aspirin cartridge, VASP, PFA-100 Col/EPI cartridge, MEA, and TEG. The first four platelet function tests are used to define HPR for aspirin and the P2Y12 inhibitor per patient. The ROC-curve derived cut-off values are used to determine HPR per platelet function test (Table 2). Depending on which P2Y12 inhibitor the patient is showing HPR, the APT is adjusted to a stronger P2Y12 inhibitor (Table 3). Until now in our experience, no patient using ticagrelor showed HPR. Patients in whom medication is adjusted are invited to our ST clinic for a second time. Platelet function tests are repeated to check whether the medication switch has overcome HPR. If the patient is still showing HPR, the APT is adjusted again when possible.

It has to be mentioned that this approach has not been validated and should be considered as clinical investigation in an attempt to improve clinical outcome in ST patients in daily practice.

## 7. Discussion

### 7.1. Platelet Function Testing Strategy

Strategies for personalized medicine can consist of platelet function testing and/or genetic testing. Genetic testing is mainly focused on CYP2C19, a liver enzyme that is involved in the metabolism of clopidogrel to its active metabolite. Especially the *2 and *3 gene variants of CYP2C19 are associated with HPR in clopidogrel-treated patients. However, in prasugrel- and ticagrelor-treated patients, no variance in drug response related to CYP2C19 gene variants is observed [75, 76]. Genetic testing is, therefore, only of limited use for patients using clopidogrel. The residual platelet function on antiplatelet treatment is the resultant of all variables, including genetic polymorphisms. Measuring this residual platelet function, therefore, gives a better estimation of the overall response of a patient to APT. Platelet function tests can also take into account the response to prasugrel and ticagrelor because also prasugrel-treated patients exhibit HPR [77–80]. Therefore, we chose the use of platelet function testing as the basis for our ST clinic to optimize APT.

Since a large proportion of ST patients exhibit HPR [24], we wanted to gain a comprehensive impression about the thrombotic state under APT of these patients. Therefore, we chose to use four platelet function tests which all measure different properties of thrombus formation. 

### 7.2. Decision Making Process for Antiplatelet Adjustment Strategies

The decision at our ST clinic to adjust APT for a patient is a clinical decision made by the cardiologist. The results of the platelet function tests are leading; however, when not conclusive, the decision is supported by clinical characteristics, comedication, and genetic data of the patient. At the same time, these items can also influence the switch to a particular P2Y12 inhibitor.

#### 7.2.1. Results of the Platelet Function Tests

Before interpreting the results of the platelet function tests, the compliance of the patient to the APT should be established, because noncompliance can negatively influence the results of the platelet function tests. 

HPR for aspirin therapy is defined when two out of three tests (LTA with AA, VerifyNow aspirin, PFA col/EPI) show HPR. However, a well-accepted alternative treatment for aspirin is not available yet. A postulated mechanism for HPR for aspirin is a high platelet turnover [81]. Since aspirin is an irreversible drug with a short plasma half-life, newly formed platelets are not inhibited. This high platelet turnover is seen in certain subgroups of patients, for instance, diabetic patients. Rocca and coworkers [82] showed that diabetic patients with the fastest recovery of platelet reactivity after aspirin administration had significantly more platelet inhibition with a twice-daily aspirin dosing regimen. Twice-daily dosing of aspirin could be beneficial for certain subgroups; however, this is not supported by clinical evidence until now. Also, safety of increased aspirin administration together with the new agents prasugrel and ticagrelor is not fully elucidated. In the PLATO trial, superiority of ticagrelor was shown compared to clopidogrel. However, this superiority was not seen from the results that were obtained for the PLATO trial in North America, were higher standard aspirin doses are used [67]. Accordingly, the U.S. Food and Drug Administration has recommended not to exceed aspirin dosing of 100 mg combined with ticagrelor [83]. Altogether, HPR for aspirin is mainly taken into account when discordant tests results are shown for the P2Y12 inhibitor with adjustment of the P2Y12 inhibitor as result. Otherwise, HPR for aspirin is accepted.

To determine whether the patient exhibits HPR for one of the P2Y12 inhibitors, at least two out of three tests (LTA with ADP, VerifyNow P2Y12, VASP) have to show HPR. When the results of two or three platelet function tests show NPR, the patient is responding well to its P2Y12 inhibitor and no adjustment of APT is needed. When all three platelet function tests show HPR, there is an urgent need for the patient to change APT. When discordant test results are obtained with two tests showing HPR, several considerations have to be taken into account to warrant adjustment of APT (Table 4).

#### 7.2.2. P2Y12 Inhibitor Adjusting Strategies

When a switch from clopidogrel to a stronger P2Y12 inhibitor is needed, our basis strategy is to switch to prasugrel over ticagrelor. Both are stronger P2Y12 inhibitors than clopidogrel, but prasugrel has the advantage of a daily dosing scheme. As noncompliance to APT and thus early discontinuation, is a major risk factor for ST, the daily dose of prasugrel will be easier to persist for the patient than the twice-daily dose of ticagrelor. 

Also in patients treated with a maintenance dose of prasugrel, HPR is seen. HPR on prasugrel is less seen (3–21%) [78–80] than HPR in clopidogrel-treated patients (up to 30%). Although it is unknown whether overcoming HPR on prasugrel will improve clinical outcome, we prefer to achieve NPR in all our ST patients. Therefore, when a patient shows HPR on prasugrel 5 mg, we switch to prasugrel 10 mg. And when HPR on prasugrel 10 mg is exhibited by the patient, we switch to ticagrelor (Table 3). Some examples of switching to prasugrel 20 mg are described in the literature [84, 85]; however, the use of prasugrel 20 mg is off-label and therefore not favorable to be used in the clinic. Because ticagrelor can reduce platelet function to a very low level [86], and a direct pharmacodynamic comparison of ticagrelor and prasugrel showed lower platelet function levels for ticagrelor [87], we switch patients with HPR for prasugrel 10 mg to ticagrelor. We are aware that this choice, of prasugrel over ticagrelor and mainly only using ticagrelor when HPR on prasugrel is seen, is our personal preference which is questionable. 

#### 7.2.3. Clinical Characteristics

Despite that our basic strategy is to switch from clopidogrel to prasugrel, prasugrel cannot be prescribed to every patient. Patients with a cerebrovascular accident or a transient ischemic attack as comorbidity in their medical history do have an contraindication for prasugrel [66], and therefore it is preferred to switch APT to ticagrelor. This consideration also has to be taken when all three platelet function tests show HPR. 

Further on, clinical characteristics as diabetes mellitus or an increased bleeding risk are weighted in decision making. For a patient with HPR for clopidogrel in combination with diabetes, the APT switch to prasugrel is preferred. When the patient has an increased bleeding risk alongside HPR for clopidogrel, one might consider not to change APT or to switch to prasugrel 5 mg.

#### 7.2.4. Comedication

From coadministration of amlodipine and the proton pomp inhibitors (PPI) omeprazole and esomeprazole, it is known that these drugs affect clopidogrel efficacy [88–90]. Therefore, the first choice is to replace the PPI or amlodipine and to test the patient again for HPR when possible. Once the patient does still exhibit HPR, the APT is adjusted to a stronger P2Y12 inhibitor. When it is not possible to replace the used PPI or amlodipine, APT of the patient is immediately switched to a stronger P2Y12 inhibitor. 

Protease inhibitors used in the treatment of patients with human immunodeficiency virus can influence the efficacy of prasugrel by inhibiting the liver enzyme CYP3A4 and CYP3A5 [91], an enzyme that is important for the conversion of prasugrel to its active metabolite [92]. The advice is to switch to ticagrelor, which is a direct-acting drug, and to repeat the measurement for HPR. 

#### 7.2.5. Genetics

Genotyping for CYP2C19 *2/*3 is performed for all patients who visited our ST clinic. Since we decided to base our decision making mainly on the results of the platelet function tests, the results of genotyping are only taken into account when a patient shows discordant results for the platelet function tests performed. A heterozygote or homozygote genotype for CYP2C19 *2/*3 will prompt the decision to switching the APT to a stronger P2Y12 inhibitor. 

#### 7.2.6. No Platelet Function Tests Available

When no platelet function tests are available in the clinic, and in addition also no genotyping for CYP2C19, one might consider to switch every patient presenting with a ST to prasugrel. This will decrease the number of patients with HPR and the risk for recurrent atherothrombotic events. Despite some patients will still exhibit HPR on prasugrel, we think that the use of ticagrelor as first choice is not preferable because of the twice-daily dose and the high number of patients suffering from dyspnea (clinical observation), which will not contribute to the compliance of ST patients to their APT. Beside this consideration, the choice for the use of prasugrel or ticagrelor will also be dependent on the clinical characteristics and comedication of the patient as mentioned above. The increased bleeding risk which comes along with the use of these agents should still be considered by the clinician. 

## 8. Future

The counterpart of achieving efficient levels of platelet inhibition with P2Y12 inhibitors is the increased risk of bleeding events. However, the occurrence of bleeding events is less frequent than thrombotic events and data that suggest an association between enhanced platelet inhibition and bleeding events is limited. Two recent studies showed an association of enhanced platelet inhibition caused by clopidogrel and major bleeding events [93, 94]. These studies are the first to suggest a “therapeutic window” for the use of clopidogrel, defined by platelet function testing, to prevent bleeding and thrombotic complications. Campo and coworkers confirmed the presence of a therapeutic window and suggested an optimal window with the use of the VerifyNow system between 86 and 238 PRU [95]. With the advent of the stronger P2Y12 inhibitors prasugrel and ticagrelor and their increased bleeding risk compared to clopidogrel, this therapeutic window is becoming more important. Therefore, when a clear therapeutic window for P2Y12 inhibitors is established, it should become a central tool in the practice of personalized APT strategies based on platelet function testing. 

Personalized medicine for APT in patients after PCI with stent implantation and especially ST patients is in its infancy. Ongoing trials have to reveal more insight into the use of platelet function testing and which APT switching strategies to use for personalized medicine after PCI. 

The purpose of the DANTE study (dual antiplatelet therapy tailored on the extent of platelet inhibition, NCT00774475) is to evaluate the efficacy of a tailored clopidogrel therapy in patients with UA/NSTEMI undergoing PCI with stent implantation and with a documented HPR measured by platelet function testing. Patients are randomized to either 75 mg clopidogrel or 150 mg clopidogrel daily dose.

A second study that examines the relation between platelet function testing and personalized medicine is the ARCTIC study (assessment with a double randomization of (1) a fixed dose versus a monitoring-guided dose of aspirin and clopidogrel after DES implantation and (2) treatment interruption versus continuation, 1 year after stenting) [96]. The first randomization arm of the ARCTIC study aims to prove superiority of monitoring with platelet function testing with dose adjustment as compared to the APT treatment according to international guidelines to reduce the primary end point after 1 year after DES implantation. Three important questions regarding personalized medicine strategies will be investigated, as the study incorporates monitoring with platelet function testing versus standard treatment, according to the current international guidelines, dose adjustment strategies are doubling aspirin dose and doubling clopidogrel dose or switching to prasugrel to prevent thrombotic events, and patients with very low on-treatment platelet reactivity are switched backwards to clopidogrel 75 mg daily dose, if on prasugrel 10 mg or clopidogrel 150 mg daily dose, to prevent bleeding events. The disadvantage of the study is the exclusion of patients with STEMI due to the randomization process, which is a high-risk group to be expected to benefit from personalized APT strategies.

Another interesting study is the TRILOGY-ACS Platelet Function Substudy (the targeted platelet inhibition to clarify the optimal strategy to medically manage acute coronary syndromes) which will provide more insight into the value of platelet function and genetic testing as a tool for personalized medicine [97]. Medically managed non-STEMI patients are randomized to treatment with either prasugrel and aspirin or clopidogrel and aspirin. In a subgroup of patients platelet function testing for aspirin and P2Y12 inhibitors at several time points throughout the study and pharmacogenetic testing is performed. Although the study does not switch APT based on the results of platelet function or genetic testing and only include medically managed non-STEMI patients, synchronous testing of the platelet function and pharmacogenetics can reveal insights in which a single method or a combination is optimal to select the patients that would benefit from personalized medicine. 

## 9. Conclusion

The personalized medicine approach to tailor APT for patients with cardiovascular disease is in development. Where low-risk groups do not seem to benefit from this new strategy, ongoing studies need to provide strategies for platelet function and/or genetic testing for personalized medicine with clinical benefit for high-risk patients after PCI with stent implantation. 

The personalized APT approach we have chosen to combat HPR based on platelet function testing in ST patients is challenging to replicate by other centers. Therefore, the development of a model that helps the cardiologist to navigate through all the different components of platelet function and genetic testing, clinical characteristics, co-medication, and the different antiplatelet drugs to prevent atherothrombotic and bleeding events is needed. This model could also serve for personalized APT strategies in patients without ST after PCI.

## Figures and Tables

**Table 1:** Definitions of stent thrombosis according to the level of documentation and timing.

Category	Description	
Level of documentation		

	Angiographic confirmation	
	(1) The thrombus	
	(i) Originates in the stent or within 5 mm proximal or distal from the stent	
	(ii) Can be either occlusive or nonocclusive	
Definite	(2) And is accompanied within a 48-hour time window with:	
	(i) Acute onset of ischemic symptoms in rest	
	(ii) New ECG changes suggesting for acute ischemia	
	(iii) Typical rise and fall in cardiac biomarkers (defined as for spontaneous MI)	
	Pathological confirmation	
	(1) Evidence of recent thrombus within the stent determined at autopsy	
	(2) Examination of tissue retrieved from thrombectomy	

Probable	Clinical definition
	(1) Any unexplained death within 30 days after PCI with stent implantation
	(2) Irrespective of the time after PCI with stent implantation, any MI that is related to documented acute ischemia in the territory of the implanted stent without angiographic confirmation and in the absence of any other obvious cause

Possible	Clinical definition
	Any unexplained death from 30 days after PCI with stent implantation until the end of trial followup

Timing after PCI with stent implantation		

Acute	0 to 24 hours	Early onset
Subacute	>24 hours to 30 days	
Late	>30 days to 1 year	Late onset
Very late	>1 year	

ECG: electrocardiogram; PCI: percutaneous coronary intervention; MI: myocardial infarction.

**Table 2:** Cut-off values to determine high on-treatment platelet reactivity.

Platelet function test	Cut-off value	Reference
P2Y12 inhibitor		

VerifyNow P2Y12	>235 PRU	Breet et al., [7]
Light transmittance aggregometry		
5 *μ*mol/L ADP	>42.9%	Breet et al., [7]
20 *μ*mol/L ADP	>64.5%	Breet et al., [7]
VASP assay	>50%	Bonello et al., [74]

Aspirin		

VerifyNow aspirin	>454 ARU	Breet et al., [7]
Light transmittance aggregometry		
0.5 mg/mL arachidonic acid	>20.0%	Gum et al., [50]
PFA-100 Col/EPI	<193 seconds	Frelinger et al., [46]

ADP: adenosine diphosphate; ARU: aspirin reaction unit; Col/ADP: collagen/ADP; Col/EPI: collagen/epinephrine; PFA-100: platelet function analyzer-100; PRU: P2Y12 reaction units; VASP: vasodilator-stimulated phosphoprotein.

**Table 3:** Model of adjusting strategies for antiplatelet therapy while having HPR.

P2Y12 inhibitor adjusting strategies	
From	To
Clopidogrel 75 mg 1dd1	Prasugrel 10 mg 1dd1 or Prasugrel 5 mg 1dd1 (when <60 kg and/or >75 years)
Prasugrel 5 mg 1dd1	Prasugrel 10 mg 1dd1
Prasugrel 10 mg 1dd1	Ticagrelor 90 mg 2dd1

**Table 4:** Overview on how to decide to optimize personalized medicine for P2Y12 inhibitors per patient.

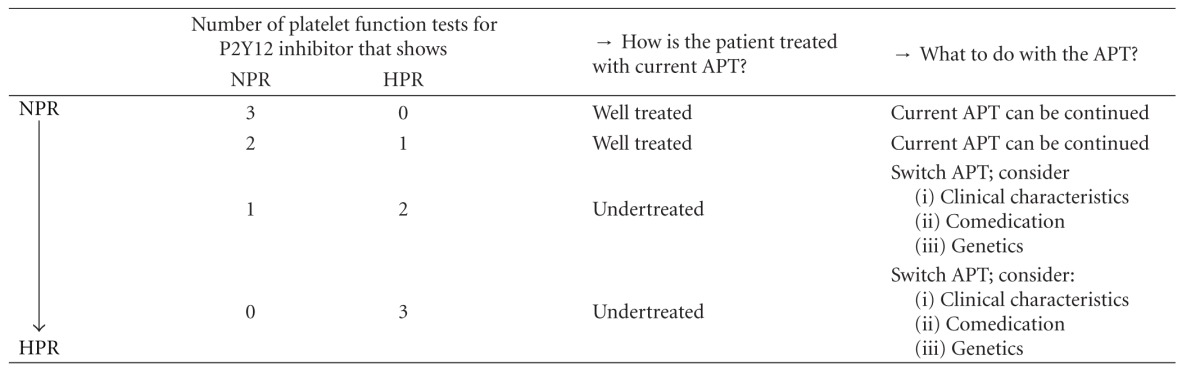

NPR: normal on-treatment platelet reactivity; HPR: high on-treatment reactivity; APT: antiplatelet therapy.
